# Tannic Acid Induces the Mitochondrial Pathway of Apoptosis and S Phase Arrest in Porcine Intestinal IPEC-J2 Cells

**DOI:** 10.3390/toxins11070397

**Published:** 2019-07-09

**Authors:** Ji Wang, Haisi Xiao, Yuanyuan Zhu, Shuiping Liu, Zhihang Yuan, Jing Wu, Lixin Wen

**Affiliations:** 1 Laboratory of Animal Clinical Toxicology, Department of Clinical Veterinary Medicine, College of Veterinary Medicine, Hunan Agricultural University, Changsha 410128, China; 2 Hunan Collaborative Innovation Center of Animal Production Safety, Changsha 410128, China

**Keywords:** anti-nutritional factor, apoptosis, IPEC-J2 cell line, tannic acid

## Abstract

The presence of tannic acid (TA), which is widely distributed in plants, limits the utilization of non-grain feed. Illustrating the toxicity mechanism of TA in animals is important for preventing poisoning and for clinical development of TA. The aim of the present study was to evaluate the toxic effects and possible action mechanism of TA in porcine intestinal IPEC-J2 cells, as well as cell proliferation, apoptosis, and cell cycle. We investigated the toxic effects of TA in IPEC-J2 cells combining the analysis of TA-induced apoptotic responses and effect on the cell cycle. The results revealed that TA is highly toxic to IPEC-J2 cells. The stress-inducible factors reactive oxygen species, malondialdehyde, and 8-hydroxy-2′-deoxyguanosine were increased in response to TA. Furthermore, TA suppressed mitochondrial membrane potential, reduced adenosine triphosphate production, and adversely affected B-cell lymphoma-2 (Bcl-2), Bcl-2-associated X protein, caspase-9, caspase-3, cytochrome *c*, cyclin A, cyclin-dependent kinases, ataxia-telangiectasia mutated, and P53 expression in a dose-dependent manner. We suggest that TA induces the mitochondrial pathway of apoptosis and S phase arrest in IPEC-J2 cells.

## 1. Introduction

Anti-nutritional factors are metabolites synthesized by plants and are known to exert effects opposite to those of the optimum nutrition. In agriculture, these substances can reduce feed intake, interfere with nutrient digestion and absorption, and induce poisoning in livestock, which limits the utilization of plant-based feedstuffs. Tannic acid (TA) is a natural product of metabolism in plants and is one of the anti-nutritional factors, found in a variety of forages, such as soybean, sunflower seed, faba bean, and alfalfa [1]. 

Tannic acid is a hydrolysable phenolic molecule, containing a glucose unit and gallic acid esterified to it. As a natural bioactive extract, multifaceted biological functions of TA have been reported in in vivo and in vitro studies. On one hand, a large number of studies have focused on the positive effects of TA in animal production. For example, it has been demonstrated that TA has an antioxidant ability, which can be used as a meat preservative to maintain quality during storage [2]. Studies have also shown that TA inhibits the growth of various bacteria by disrupting the bacteria cell wall and activating cytokine production [3,4]. In addition, there is evidence that TA has hepatitis C virus (HCV) antiviral activity in cell culture and can be developed as a relatively inexpensive adjuvant [5]. On the other hand, the toxicity of TA has also been receiving considerable attention. It has been found that oral and parenteral exposure to TA induces lesions in the renal, liver, and gastrointestinal tissues and suppresses immune system functions in animals. An acute toxicity study in six adult merino ewes showed that 1 g/kg/animal/day of TA induced abomasum superficial mucosal erosion, hemorrhage, submucosal edema, midzonal or periacinar coagulative necrosis in the liver, and focal tubular vacuolation and necrosis in the kidneys [6]. However, the adverse effects of TA in animals are closely related to the dosage and actuation duration. Tannic acid has been proved to be lethal to bovines after daily dosage of 50 g for 16 days, but a daily dose of 25 g for 28 days did not exert any adverse effects [7].

IPEC-J2 is an epithelial cell line that was isolated from the mid-jejunum of neonatal piglet in 1989 by Helen Berschneider [8]. This cell line is being increasingly used to simulate the porcine gastrointestinal tract. To the best of our knowledge, there is no information on the effects of TA in the porcine gastrointestinal tract. The aim of the present study was to evaluate the toxic effects and possible action mechanism of TA in IPEC-J2 cells, as well as cell proliferation, apoptosis, and cell cycle. 

## 2. Results

### 2.1. TA Induced Cytotoxic Effect in IPEC-J2 Cells

**Figure 1:**
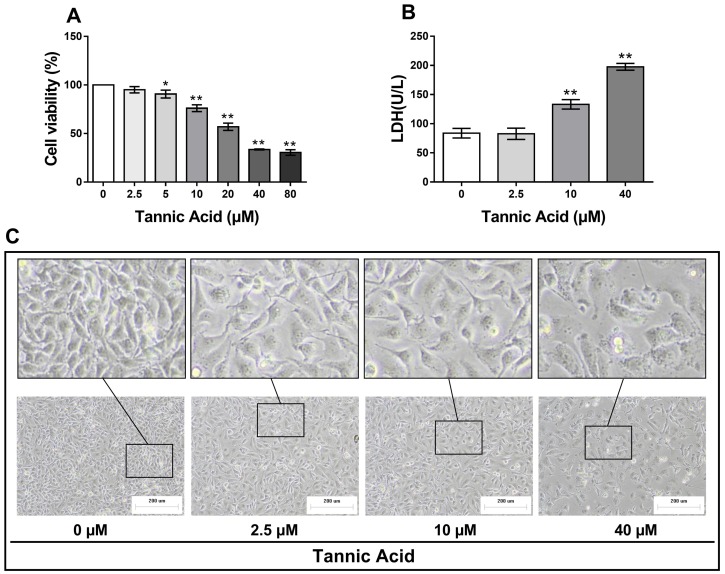
Tannic acid (TA) exerted cytotoxic effect in IPEC-J2 cells. The cells were treated with or without TA at different doses for 24 h. (**A**) TA induced a typical dose-dependent inhibition of IPEC-J2 cell proliferation; (**B**) the activity of lactate dehydrogenase (LDH) was increased after treatment with 10 and 40 μM TA; (**C**) the cell morphology was changed significantly and growth was decreased after TA treatment (magnification 100×, Scale bar = 200 μm). The results are expressed as mean ± SD; *n* = 6 for each group. ** *p* < 0.01 and * *p* < 0.05 are considered significantly different from the control.

The cytotoxic effects of TA on IPEC-J2 cells were examined using the 3-[4,5-dimethylthiazol-2-yl]-2,5-diphenyltetrazolium bromide (MTT) assay. As shown in Figure 1A, in IPEC-J2 cells, treatment with TA at different doses (0, 2.5, 10, 20, 40, and 80 μM) for 24 h induced a typical dose-dependent inhibition of cellular proliferation. The cell viability decreased to 95 ± 5.63%, 76.1 ± 6.03% and 33.6 ± 0.83% after 2.5, 10, and 40 μM TA treatment. The IC50 value was calculated as 28.47 ± 5.79 μM. Based on this dose-dependent effect, 0, 2.5, 10, and 40 μM TA were selected as the dose range for the subsequent experiments. After 24 h of treatment with 10 and 40 μM TA, IPEC-J2 cells exhibited a significantly higher level of lactate dehydrogenase (LDH) in culture medium than that of the control group, indicating severe intestinal damage (Figure 1B). As shown in Figure 1C, in vitro, the cell morphology was changed significantly and growth was decreased after TA treatment.

### 2.2. TA Induced Oxidative Stress and DNA Damage in IPEC-J2 Cells

**Figure 2:**
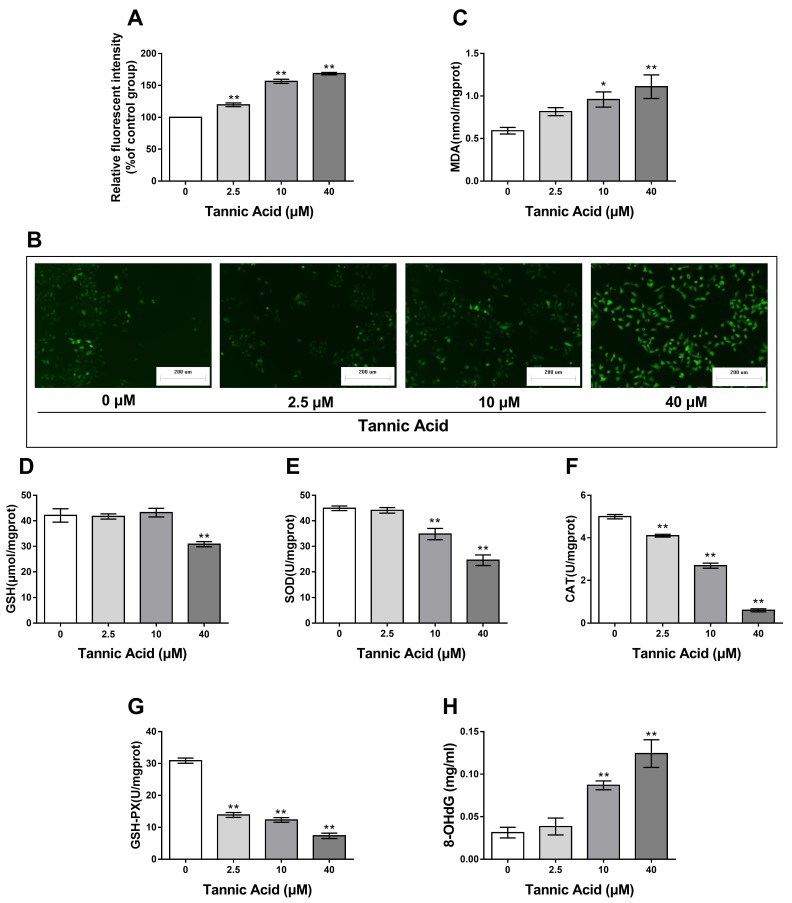
Effects of TA on oxidative stress and deoxyribonucleic acid (DNA) damage in IPEC-J2 cells. The cells were treated with or without TA at different doses for 24 h. (**A**,**B**) oxygen species (ROS) generation in IPEC-J2 cells increased after treatment with TA (magnification 100×, Scale bar = 200 μm); (**C**,**D**) TA treatment increased malondialdehyde (MDA) and decreased glutathione (GSH) content in IPEC-J2 cells; (**E**–**G**) TA promoted a typical dose-dependent reduction in superoxide dismutase (SOD), catalase (CAT), and glutathione peroxidase (GSH-PX) activities in IPEC-J2 cells; (**H**) 8-hydroxy-2′ -deoxyguanosine (8-OHdG) level in cultural media of IPEC-J2 cells was increased following the treatment with TA. The values are the mean ± SD of three independent experiments. ** *p* < 0.01 and * *p* < 0.05 are considered significantly different from the control.

Oxidative stress in cells is indicated by a high level of reactive oxygen species (ROS), which is caused by decreased activity of antioxidant enzymes and decreased antioxidant capacity indicated by glutathione (GSH). Over-accumulation of ROS damages the cell membrane components, including lipids, indicating increased production of malondialdehyde (MDA). As shown in Figure 2E–G, when the cells were treated with TA for 24 h, the activity of superoxide dismutase (SOD), catalase (CAT), and glutathione peroxidase (GSH-PX) was significantly decreased in a dose-dependent manner with concomitant decrease in the GSH level for 40 µM TA (Figure 2D). Significant ROS production (Figure 2A,B) was observed, followed by a significant increase in the MDA level (Figure 2C). These results demonstrated that TA can induce oxidative damage in IPEC-J2 cells.

To investigate the changes in DNA damage induced by TA, the content of 8-hydroxy-2′-deoxyguanosine (8-OHdG) was evaluated. Expectedly, IPEC-J2 cells exhibited a considerable increase in 8-OHdG after TA treatment. The level of DNA damage after TA (20–40 μM) treatment was significantly increased compared with that in the control (*p* < 0.01). These results confirm that TA induced DNA damage in IPEC-J2 cells (Figure 2H).

### 2.3. TA Induced Mitochondrial Dysfunction

**Figure 3:**
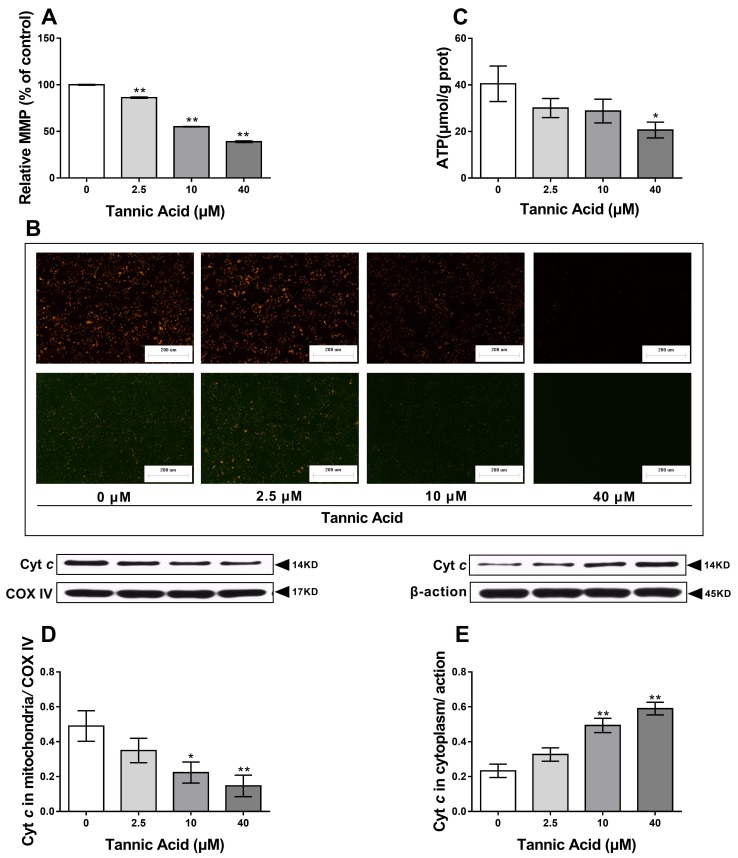
Effects of TA on mitochondrial dysfunction in IPEC-J2 cells. The cells were treated with or without TA at different doses for 24 h. (**A**,**B**) TA decreased mitochondrial membrane potential (MMP) in a dose-dependent manner in IPEC-J2 cells (magnification 100×, Scale bar = 200 μm); (**C**) 40 μM TA reduced the production of adenosine triphosphate (ATP) in IPEC-J2 cells; (**D**,**E**) cytochrome *c* (Cyt *c*) was released from the mitochondria into the cytoplasm in IPEC-J2 cells treated with TA. The data are mean ± SD of three different experiments. ** *p* < 0.01 and * *p* < 0.05.

To examine whether TA can induce mitochondrial dysfunction in IPEC-J2 cells, the mitochondrial membrane potential (MMP) and ATP levels were evaluated after treatment with TA for 24 h. TA decreased the MMP in a dose-dependent manner, which was confirmed by the fluorescence images (Figure 3A,B). To further analyze the mitochondrial function, the total ATP level was determined. Consistent with the effect of TA on the MMP, TA significantly inhibited the production of ATP at the concentration of 40 µM (Figure 3C). These results suggested that TA induced the damage of mitochondrial functions and integrity. As shown in Figure 3D,E, the level of Cyt *c* in mitochondrion was decreased in a TA concentration dependent manner ( Figure 3D), whereas in cytoplasm it showed the opposite trend (Figure 3E). These suggested that proapoptotic molecule cytochrome *c* (Cyt *c*) was released from the mitochondria into the cytoplasm, suggesting TA-induced apoptosis was associated with the mitochondria.

### 2.4. TA Induced the Mitochondrial Pathway of Apoptosis in IPEC-J2 Cells

**Figure 4:**
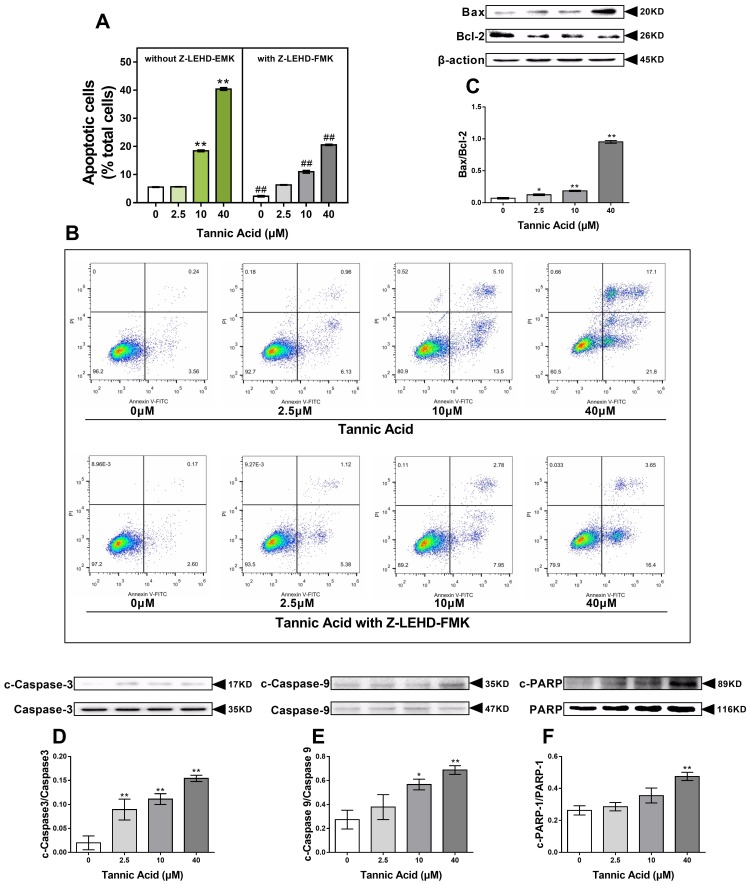
Effects of TA on the mitochondrial pathway of apoptosis in IPEC-J2 cells. The cells were treated with or without TA at different doses for 24 h. (**A**,**B**) 10 and 40 μM TA induced apoptosis in IPEC-J2 cells, and was significantly counteracted by caspase-9 inhibitor (Z-LEHD-FMK) treatment; (**C**–**F**) the ratio of Bcl-2-associated X protein (Bax)/B-cell lymphoma-2 (Bcl-2) and cleavage of caspase-3, caspase-9, and poly [ADP-ribose] polymerase 1 (PARP-1) were enhanced by treatment with a high dose of TA. The data are mean ± SD of three different experiments. ** *p* < 0.01 and * *p* < 0.05.

Mitochondrial dysfunction elicited by TA can cause cell apoptosis. To further evaluate whether TA causes the apoptosis of IPEC-J2 cells, we performed Annexin/ propidium iodide (PI) staining to distinguish and quantify the apoptotic cells. As shown in Figure 4A,B, in cells treated with TA, there was an accumulation of cells in the apoptotic phase. In cells treated with 10 and 40 μM TA, the apoptosis rate (18.47% and 40.43%, respectively) was comparatively higher than that in the untreated cells (5.57%, *p* < 0.01), showing a dose-dependent induction of apoptosis. Tannic acid-induced apoptosis was significantly counteracted by caspase-9 inhibitor (Z-LEHD-FMK) treatment. Our result suggested that the mitochondrial pathway of apoptosis is one of the important mechanisms of TA-induced apoptosis.

As regulatory proteins of apoptosis, we examined the level of B-cell lymphoma-2 (Bcl-2) and Bcl-2-associated X protein (Bax) in IPEC-J2 cells. Upon treatment with TA, the expression of the proapoptotic protein Bax was significantly increased and that of the anti-apoptotic protein Bcl-2 was decreased, and concomitantly, the ratio of Bax/Bcl-2 was increased in a dose-dependent manner (Figure 4C). As a downstream molecule of Cyt *c*, caspase-9 was cleaved and activated, indicating the mitochondrial pathway of apoptosis. After 24 h of TA treatment, the cleaved caspase-9 level was significantly increased compared with that in the control group (Figure 4E). As a key executioner of apoptosis, caspase-3 cleaved poly [ADP-ribose] polymerase 1 (PARP-1) into 89 kDa fragments, which could suppress the DNA mismatch repair system, causing apoptosis. The cleavage of caspase-3 was significantly enhanced in IPEC-J2 cells induced by TA in a dose-dependent manner, followed by an increase in the expression of cleaved-PARP-1 (Figure 4D,F).

### 2.5. TA Induced the S Phase Arrest of IPEC-J2 Cells

**Figure 5:**
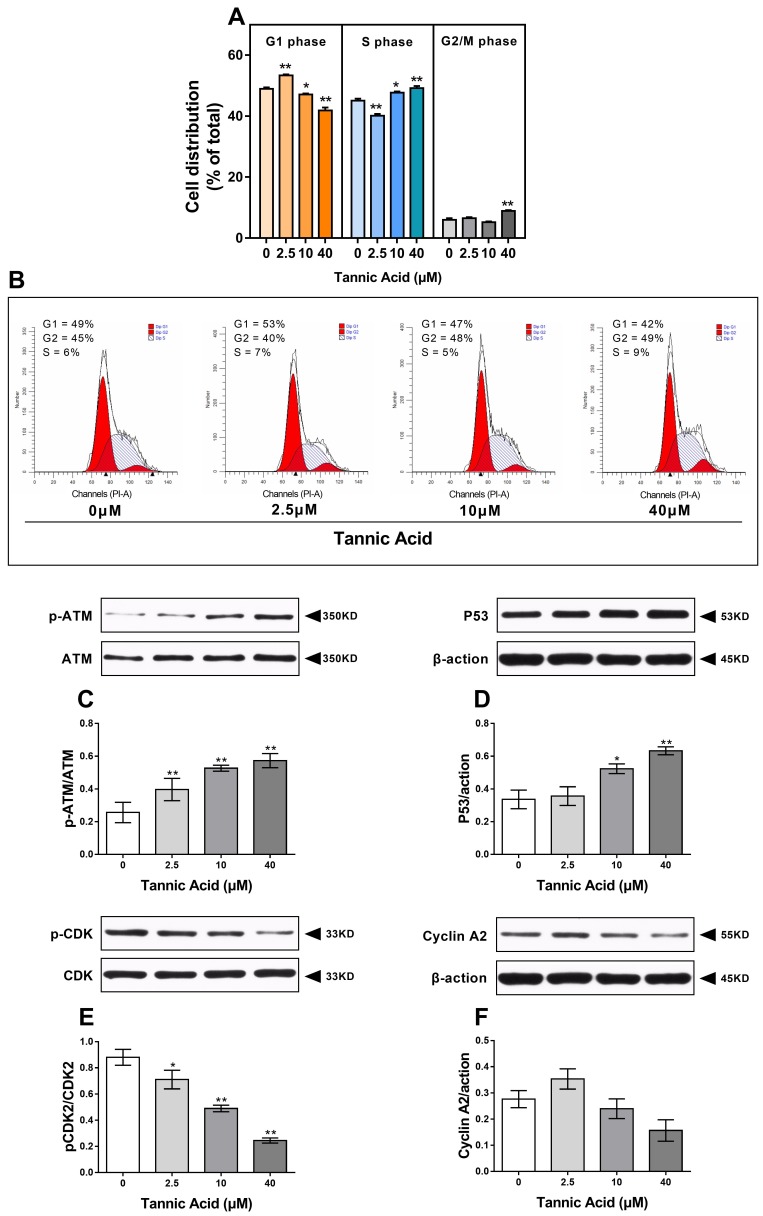
Effects of TA on the cell cycle in IPEC-J2 cells. The cells were treated with or without TA at different doses for 24 h. (**A**,**B**) The cells treated with 10 and 40 μM TA accumulated in the S phase with a decrease in the percentage of cell population in the G1 phase; (**C**) the phosphorylation of ataxia–telangiectasia mutated (ATM) was significantly increased in IPEC-J2 cells by TA treatment; (**D**) the expression of protein 53 (P53) was increased in a dose-dependent manner; (**E**) TA treatment inhibited the phosphorylation of cyclin-dependent kinases 2 (CDK2); (**F**) the expression of cyclin A2 peaked after treatment with 2.5 μM TA. The data are mean ± SD of three different experiments. ** *p* < 0.01 and * *p* < 0.05.

We analyzed cell distribution by PI staining. As shown in Figure 5A,B, the cells treated with 10 and 40 μM TA accumulated in the S phase with a decrease in the percentage of the cell population in the G1 phase. The percentage of cells in the S phase was higher in cells treated with 10 and 40 μM TA (47%, *p* < 0.05 and 49%, *p* < 0.01, respectively) compared with that in the control group, showing a dose-dependent S phase arrest in IPEC-J2 cells.

To investigate molecular mediators in TA-induced S phase arrest and apoptosis, we assessed the changes in the ataxia–telangiectasia mutated (ATM)-protein 53 (P53)-cyclin-dependent kinases 2 (CDK2)/cyclin A2 signaling pathway by western blotting. The results (Figure 5C) showed that the phosphorylation of ATM was significantly increased in IPEC-J2 cells treated with TA compared with that in the control cells. Consistent with the results of ATM, 24 h after TA treatment, the expression of P53 increased in a dose-dependent manner (Figure 5D). It has been reported that the CDK2/cyclin A complex formed by the binding of CDK2 with cyclin A is regulated during the S-phase of the cell cycle [9]. As shown in Figure 5E, we observed that TA treatment inhibited the phosphorylation of CDK2. In addition, the expression of cyclin A2 peaked when the dose of TA was 2.5 μM, but it was not statistically significant (Figure 5F).

## 3. Discussion

Tannic acid exhibits anti-proliferative characteristics in a broad range of tissues by targeting multiple signaling pathways. Previous studies have demonstrated the negative effects of TA on cell viability. It has been shown that the viability of neutrophils significantly decreased after 4 h of TA treatment [10]. This is similar to the results observed in the digestive cells of freshwater mussel *Unio tumidus* treated with TA [11]. In the present study, the cytotoxicity of TA at different doses was evaluated 24 h after treatment. The results clearly showed that TA at doses higher than 2.5 μM can induce serious cytotoxicity in IPEC-J2 cells. Compared with those of the control group, the release of LDH and morphology of treated cells further verified the cytotoxicity of TA. 

Various mechanisms of TA-mediated cytotoxicity have been discussed. However, the mechanism underlying oxidative-stress-mediated apoptosis has not been reported. To the best of our knowledge, our study is the first to determine the biochemical parameters of oxidative stress. Reactive oxygen species are oxidizing agents and include superoxide anion (O^2−^), hydroxyl radicals (OH^-^), and some substances generating free radicals such as H_2_O_2_ [12]. Under healthy conditions, ROS are mainly produced by cell metabolism, and they function as signaling molecules attacking pathogenic microorganisms and regulating cell growth [13,14]. Excessive ROS are eliminated by anti-oxidative enzymes including SOD, GSH-PX, and CAT and other non-enzymatic antioxidants [15]. When antioxidant defenses are disrupted, the over-accumulation of ROS due to oxidative stress leads to significant damage of cellular components both within the mitochondria and cells as determined by increased MDA level, which is a main product of lipid peroxidation [16]. As presented in our study, TA induced oxidative stress in the IPEC-J2 cell model. Furthermore, it was highly toxic to the cells, with significant toxicity at 10 μM dose as indicated by decreased activity of CAT, GSH-PX, and SOD and increased level of ROS and MDA in a TA dose-dependent manner. Similar results have been previously reported [17].

It is known that excessive ROS results in an attack not only on lipid but also on other cellular components including nucleic acids, which are extremely sensitive to oxidation [18]. DNA damage in IPEC-J2 cells was observed after TA treatment as determined by the level of 8-OHdG in culture medium, which is a widely used biomarker of oxidative stress [19]. In the present study, the ELISA results revealed that that treatment with 10 μM resulted in apparent DNA damage in IPEC-J2 cells, suggesting that DNA damage is likely to be one of the response mechanisms of IPEC-J2 cells to TA. It noteworthy that TA as a polyphenolic compound exhibits anti-oxidative properties in animals. It has been demonstrated that TA strengthens the potential of antioxidant defense and is beneficial for the suppression of oxidative stress by increasing the activity of CAT and GSH-PX [20]. These polyphenols can behave as both antioxidants and pro-oxidants depending on the concentration and free radical source [11]. Another study reported that polyphenols displayed a pro-oxidant activity in the presence of Cu^2+^ or Fe^3+^. This kind of reactions can induce lipid peroxidation and DNA strand breaks [21]. There is also a possibility that TA chelates with ions and binds to proteins and cause precipitation, increasing their toxicity [22]. The results of the present study suggest that TA results in oxidative stress, damaging the digestive tract in a pig.

The mitochondria play a central role in the regulation of cellular bioenergetics, regulating ATP synthesis, calcium homeostasis, apoptosis, and ROS production. Excessive ROS in the cytoplasm can damage mitochondrial functional with various mechanisms, including the induction of oxidative mitochondrial DNA damage, increase in excessive calcium ion influx, inhibition of key phosphatases, and induction of key kinases and transcription factors [23]. On one hand, mitochondrial DNA damage induced by ROS leads to defective complex I or III, which can result in increased electron reduction of O_2_ to form superoxide [24,25,26]. On the other hand, excessive ROS induce lipid peroxidation in the mitochondrial membrane, decreasing the MMP [27]. Impairment of mitochondrial dynamics and morphology further lead to the accumulation of ROS, resulting in a vicious cycle between mitochondria and ROS [28]. The decreased MMP and ATP level suggested that TA-induced ROS generation damages the mitochondria morphometry and function. 

Normally, the mitochondrial inner membrane is impermeable, but permeability transition can be initiated by oxidative stress, nitric oxide, calcium overload, or apoptotic protein upregulation. Mitochondrial membrane permeability transition results in the activation of the caspase cascade via the release of Cyt *c* and apoptosis-inducing factors, causing apoptosis or programmed cell death. Mitochondrial membrane permeability transition is a critical aspect in apoptosis [16]. Our results showed that ROS induced by TA disrupts the MMP and results in mitochondrial permeability transition, which caused Cyt *c* release into the cytoplasm. Members of the Bcl-2 family can be classified into anti-apoptosis Bcl-2-like proteins and proapoptotic Bax-like members [29]. In healthy cells, Bax resides in the cytoplasm and translocated constantly to the mitochondria, where the anti-apoptosis Bcl-2 proteins bind to Bax and translocate back into the cytoplasm, and thereby stabilizing the inactive Bax conformation [30]. In response to apoptosis, Bax translocates and oligomerizes to the mitochondrial outer membrane to a canal that leads to a second mitochondria-derived activator of caspase (SMAC)/Diablo, Omi/HtrA Serine Peptidase 2 (HtrA2), and Cyt *c* escape [31,32]. When the Bax/Bcl-2 ratio ispromoted, the downstream caspase cascade response can be activated resulting in apoptosis [33]. Several studies have demonstrated that Cyt *c* is a regulatory protein that binds to the Apaf-1 protein, generating a high molecular weight protein complex, and then activates the initiator caspase-9, which is specific to the mitochondrial apoptotic pathway [34]. In the present study, TA remarkably raised the level of pro-apoptotic protein Bax and downregulated the level of anti-apoptotic protein Bcl-2. The level of Cyt *c* in the cytoplasm was evidently ascended, whereas it was opposite in the mitochondria. The caspase-9 molecule is activated by Cyt *c*. As an ultimate executor of apoptosis, caspase-3 is activated by caspase-9, leading to the cleavage of PARP-1, which is downstream of caspase-3. In the present study in IPEC-J2 cells, the results showed a TA-induced increase in caspase-3 and caspase-9, suggesting that TA mediated oxidative stress-initiated apoptosis via the mitochondrial pathways, which is confirmed by the results of flow cytometry.

Fundamental and pioneer studies have demonstrated that DNA damage might change the cell cycle processes [35,36]. In the present study, we observed that DNA damage was induced by TA treatment after 24 h. Thus, we evaluated the effect of TA on the cell cycle. The process of the cell cycle is complex and rigorous, which involves doubling of DNA and other cellular contents to duplicate a cell. The cell cycle is divided into four phases, including the G1, S, G2, and M phases. The mechanism of cell entry checkpoints determines whether the cell will proceed to division or stop. Once the cell cycle has abnormalities, the checkpoint signaling pathway is activated, immediately preventing the cell cycle to ensure the validity of DNA replication and chromosome division [37]. In the present study, after 24 h of TA treatment, the proportion of S phase cells significantly increased after treatment with TA at doses of 10 and 40 μM, whereas it was contrary at 2.5 μM. This indicated that the oxidative stress induced by TA caused DNA damage and triggered the S-phase arrest. There is evidence that excessive ROS results in DNA damage during the S phase [38]. It is similar to our results. 

The occurrence of DNA damage activates phosphoinositide 3-kinase (PI3K)-like kinases such as ATM kinase. Phosphorylated ATM activates the downstream molecule P53, which regulates numerous cellular responses, including cell cycle arrest and apoptosis. P53 is a key factor involved in the activation of cell cycle checkpoints, resulting in cell cycle arrest (G1/S, G2/M) to gain more time for DNA repair [39,40,41,42]. In our study, we observed that ATM was phosphorylated in a dose-dependent manner after TA treatment, with a concomitant increase in the expression of P53. This suggested that the ATM-P53 signaling pathway is involved in S phase arrest induced by DNA damage after TA treatment. The cyclin A/CDK2 complex particularly regulates the priming and progression of DNA synthesis [43]. Studies have reported that the inhibition of CDK2 phosphorylation could cause the accumulation of cells in the S phase [44]. However, it was converse in Hela cells; the activated CDK2 could result in S-phase arrest [45]. The results suggest that CDK2 regulates the cell cycle. In the present study, after TA treatment, analysis of the cell cycle revealed a significant increase in IPEC-J2 cells in the S-phase, accompanied by a rapid decrease in CDK2 phosphorylation and cyclin A expression. These delineate an important role of TA in activating the S-phase arrest in IPEC-J2 cells, which is mainly mediated by the ATM-P53-CDK2/cyclin A-mediated pathway. It is noteworthy that the S phase of IPEC-J2 cells was reduced after 2.5 µm TA treatment, and it might be due to the activation of the ATM-P53 signaling pathway, resulting in DNA repair and promoting the cells to enter normal cycles. 

As discussed above, TA leads to oxidative stress via the generation of ROS, induces the mitochondrial pathway of apoptosis via ROS-mediated mitochondrial dysfunction, and causes S phase arrest via ROS-mediated DNA damage in IPEC-J2 cells. However, there is a complex relationship among oxidative stress, apoptosis, and cell cycle arrest, and further research is required to elucidate the mechanisms of toxic effects of TA in vitro and in vivo.

## 4. Conclusions

In conclusion, in this study, we demonstrated the toxic effects of TA on porcine enterocytes. The results showed that TA induces oxidative stress and DNA damage via excessive generation of ROS, resulting in mitochondrial dysfunction and triggering the mitochondrial pathway of apoptosis and S phase arrest in IPEC-J2 cells. It provides an experiment-based reference for the usage of TA in animal feeds and food industry.

## 5. Materials and Methods

### 5.1. Chemicals

Tannic acid was purchased from Shanghai Macklin Biochemical Technology Co., Ltd., (Shanghai, China). MTT was obtained from Solarbio (Beijing, China). The bicinchoninic acid (BCA) protein assay kit, LDH cytotoxicity assay kit, superoxide dismutase (SOD) assay kit, catalase (CAT) assay kit, glutathione peroxidase (GSH-PX) assay kit, lipid peroxidation (MDA) assay kit, and ATP assay kit were purchased from Nanjing KeyGen Biotech. Co. Ltd (Nanjing, China). The Annexin V-FITC apoptosis detection kit and mitochondrial membrane potential assay kit were obtained from Nanjing KeyGen Biotech. Co. Ltd. The ROS content assay kit was purchased from Beyotime Biotech. Co. Ltd. (Nantong, China). Rabbit monoclonal antibody of Bcl-2, Bax, PARP-1, caspase-3, caspase-9, cytochrome *c*, P53, ATM, P-ATM. CDK, P-CDK, cyclin A2, and β-actin, and goat anti-rabbit antibody of IgG-HRP were obtained from Cell Signaling Technology (Boston, MA, USA). Z-LEHD-FMK was purchased from Abcam, Inc. (Cambridge, UK). The 8-OHdG assay kit was purchased from Elabscience Biotechnology Co., Ltd. (Wuhan, China).

### 5.2. Cell Culture

IPEC-J2 cells were obtained from BeNa Culture Collection (Beijing, China) and cultured in HyClone RPMI-1640 medium (Thermo Scientific Pierce Protein Biology, Waltham, MA, USA) containing 10% Sijiqing fetal bovine serum (Zhejiang Tianhang Biotechnology Co., Ltd., Huzhou, China), supplemented with 100 U/mL Solarbio penicillin (Beijing Solarbio Science & Technology Co., Ltd., Beijing, China). The cells were maintained in 25-cm^2^ culture flasks or 96 well (5% CO_2_, 37 °C), and cultured for 24 h before subjecting to different treatments.

For TA treatments, an appropriate amount was dissolved in double distilled water and further diluted in serum-RPMI-1640 medium to achieve the indicated final concentration (2.5, 5, 10, 20, 40, and 80 μM). Serum-RPMI-1640 medium was removed and replaced with fresh serum-RPMI-1640 medium containing different concentrations of TA for 24 h. To investigate the role of the mitochondrial pathway in TA-induced apoptosis, the cells were pre-treated with the caspase-9 inhibitor Z-LEHD-FMK (20 μM) for 30 min and then treated with fresh medium containing TA at different concentrations for 24 h.

### 5.3. Determination of Cell Viability

Cell viability was determined using the MTT assay. Briefly, 24 h after TA treatment, the treatment medium was replaced with fresh medium including MTT (0.25 mg/mL) and incubated for another 4 h. The supernatant was removed and replaced with 150 µL of dimethyl sulfoxide (DMSO) to dissolve the formazan crystals. The absorbance of the sample was measured at 490 nm using a microplate reader (Infinite^®^ M1000 Pro, TECAN, Grödig, Austria).

### 5.4. Determination of LDH Activity

To evaluate the cytotoxicity induced by TA, we measured the activity of LDH in culture media. The operations and result calculation were strictly carried out according to the protocol of kit.

### 5.5. Determination of Oxidative Stress Parameters

IPEC-J2 cells were collected and lysed to extract oxidative stress-related molecules and enzymes. The activity of GSH-PX, CAT, and SOD, and content of MDA and GSH were determined following the manufacturer’s recommendations. The result was normalized using the protein concentrations in cell lysates.

### 5.6. Determination of 8-OHdG

To test DNA damage induced by TA, we determined the content of 8-OHdG in cultural media using the ELISA kit according to the manufacturer’s protocol. The result is expressed as ng/mL.

### 5.7. Determination of ROS

The generation of intracellular ROS was evaluated using the fluorescent probe, 2′,7′-dichlorofluorescin diacetate (DCFH-DA). IPEC-J2 cells were seed in a six-well cell culture plate. After treatment with different doses of TA, the cells were incubated with DCFH-DA for 30 min and then washed with PBS. The DCF fluorescence intensity of the cells was measured with an inverted fluorescence (DMI 3000 B, Leica Microsystems Ltd., Buffalo Grove, IL, USA) and analyzed at an excitation wavelength of 488 nm and an emission wavelength of 525 nm.

### 5.8. Determination of Mitochondrial Membrane Potential

The MMP was determined using the JC-1 assay kit. Briefly, JC-1 exhibits a potential-dependent accumulation in the mitochondria, as indicated by a fluorescence emission wavelength shift from green (530 nm) to red (590 nm). In summary, the loss of MMP was indicated by a decrease in the ratio of red/green mean fluorescence intensity. After treatment, IPEC-J2 cells were collected and incubated with JC-1 for 20 min at 37 °C, and then washed with JC-1 staining buffer twice and analyzed by fluorescence spectrophotometry. 

### 5.9. Measure of ATP

To further evaluate the dysfunction of mitochondria induced by TA, we measured the production of ATP in cells. After treatments, the cells were collected and homogenized in boiling water for analysis following the protocol of kit.

### 5.10. Flow Cytometric Measurement of Apoptosis

Annexin-V-FITC/PI was used to distinguish and quantify apoptotic cells by flow cytometry. Briefly after treatment, the cells were collected and resuspended in 500 μL of 1× binding buffer, and then stained with 5 μL of Annexin V-FITC and 5 μL of PI for 15 min in dark. The percentage of apoptotic cells was analyzed by flow cytometry. The viable cells were Annexin V-/PI-, early apoptotic cells were Annexin V+/PI-, late apoptotic cells were Annexin V+/PI+, and cell debris was Annexin V-/PI+.

### 5.11. Cell Cycle Analysis

To study the effect of TA on the cell cycle, IPEC-J2 cells were treated with TA for 24 h after seeding in six-well plates. After treatment, the cells were collected and washed with PBS twice, and then fixed in 70% ethanol overnight. On the subsequent day, the cells were centrifuged, washed, and incubated with a PI solution at 37 °C for 30 min. The distribution of cells in different cell-cycle phases was analyzed from the DNA content histogram by flow cytometry.

### 5.12. Western Blotting

After treatment, IPEC-J2 cell protein was extracted with RIPA lysis buffer containing 1% phenylmethylsulfonyl fluoride. The total protein concentration was measured using the BCA assay kit. An equal amount of protein was loaded and separated by 4–12% sodium dodecyl sulfate polyacrylamide gel electrophoresis (SDS-PAGE) and transferred on to polyvinylidene difluoride (PVDF) membrane. The membranes were blocked with TBS-T buffer containing 0.2% gelatin for 1 h at 25 °C, and then incubated overnight with the primary antibodies (anti-caspase-3 antibody, anti-caspase-9 antibody, anti-Bcl-2 antibody, anti-Bax antibody, anti-cytochrome *c* antibody, anti-β-action antibody, anti-CDK2 antibody, anti-P-CDK2 antibody, anti-P53 antibody, anti-ATM antibody, anti-P-ATM antibody, anti-cyclin A2 antibody, and anti-COX IV antibody), followed by incubation with the secondary antibodies at room temperature 1 h. All bands were visualized by enhanced chemiluminescence and quantitatively analyzed using a Quantity One image densitometer. The protein levels were standardized by comparison with that of β-actin.

### 5.13. Statistical Analysis

The results are expressed as mean ± SEM of at least three independent experiments, and the analysis of data was performed by One-way ANOVA with LSD’s post hoc analysis using SPSS 20.0 statistical software (SPSS Inc., Chicago, IL, USA). Differences were considered to be statistically significant when *p* < 0.05.
